# Atherosclerotic Plaque in Patients with Zero Calcium Score at Coronary Computed Tomography Angiography

**DOI:** 10.5935/abc.20180063

**Published:** 2018-05

**Authors:** Fabíola Santos Gabriel, Luiz Flávio Galvão Gonçalves, Enaldo Vieira de Melo, Antônio Carlos Sobral Sousa, Ibraim Masciarelli Francisco Pinto, Sara Melo Macedo Santana, Carlos José Oliveira de Matos, Maria Júlia Silveira Souto, Flávio Mateus do Sacramento Conceição, Joselina Luzia Menezes Oliveira

**Affiliations:** 1 Núcleo de Pós-Graduação em Medicina da Universidade Federal de Sergipe (UFS) - São Cristóvão, SE - Brazil; 2 Centro de Pesquisas da Fundação São Lucas - Aracaju, SE - Brazil; 3 Clínica de Medicina Nuclear de Diabetes - CLIMEDI - Aracaju, SE - Brazil; 4 Departamento de Medicina - Universidade Federal de Sergipe (UFS) - São Cristóvão, SE - Brazil; 5 Centro de Ensino e Pesquisa e Laboratório de Ecocardiografia (ECOLAB) do Hospital e Fundação São Lucas - Aracaju, SE - Brazil; 6 Instituto Dante Pazzanese de Cardiologia - São Paulo, SP - Brazil; 7 Centro de Ensino e Pesquisa da Fundação São Lucas - Aracaju, SE - Brazil

**Keywords:** Cardiovascular Diseases/mortality, Plaque, Atherosclerotic, Coronary Artery Disease/diagnosis, Calcium Signaling, Coronary, Angiotomography, Risk Factors

## Abstract

**Background:**

In view of the high mortality for cardiovascular diseases, it has become necessary to stratify the main risk factors and to choose the correct diagnostic modality. Studies have demonstrated that a zero calcium score (CS) is characteristic of a low risk for cardiovascular events. However, the prevalence of individuals with coronary atherosclerotic plaques and zero CS is conflicting in the specialized literature.

**Objective:**

To evaluate the frequency of patients with coronary atherosclerotic plaques, their degree of obstruction and associated factors in patients with zero CS and indication for coronary computed tomography angiography (CCTA).

**Methods:**

This is a cross-sectional, prospective study with 367 volunteers with zero CS at CCTA in four diagnostic imaging centers in the period from 2011 to 2016. A significance level of 5% and 95% confidence interval were adopted.

**Results:**

The frequency of atherosclerotic plaque in the coronary arteries in 367 patients with zero CS was 9.3% (34 individuals). In this subgroup, mean age was 52 ± 10 years, 18 (52.9%) were women and 16 (47%) had significant coronary obstructions (> 50%), with involvement of two or more segments in 4 (25%) patients. The frequency of non-obese individuals (90.6% vs 73.9%, p = 0.037) and alcohol drinkers (55.9% vs 34.8%, p = 0.015) was significantly higher in patients with atherosclerotic plaques, with an odds ratio of 3.4 for each of this variable.

**Conclusions:**

The frequency of atherosclerotic plaque with zero CS was relatively high, indicating that the absence of calcification does not exclude the presence of plaques, many of which obstructive, especially in non-obese subjects and alcohol drinkers.

## Introduction

Coronary artery disease (CAD) are the leading cause of death in the world, including in Brazil. Many methods for CAD diagnosis, risk stratification of patients and indication of revascularization are currently available.^1^

One of the greatest challenges of routine cardiology practice is to determine the best method to detect subclinical CAD. Coronary computed tomography angiography (CCTA) is a predominantly anatomical test with excellent diagnostic accuracy in detecting obstructive and nonobstructive lesions as compared with coronary angiography, which is considered the gold standard method for this purpose. Also, CCTA may provide relevant information regarding atheroma composition according to radiological density.^2^^,^^3^

The role of coronary calcification, identified by calcium score (CS), used for classification of patients into a higher risk for cardiovascular events, is well known. Although individuals with a zero CS may also have atherosclerotic plaques,^4^^,^^5^ its presence has not been associated with increased risk for future cardiovascular events.^4^

Nevertheless, despite these results reported in international studies, we have not found Brazilian studies published on this specific subject. In fact, studies’ results may not be replicated in different sociodemographic or anthropometric contexts, or even in different healthcare conditions. Reproducibility of a study is a *sine qua non* for an extensive validation of its results.

Therefore, the main aim of this study is to evaluate the frequency of coronary atherosclerotic plaque, its degree of obstruction and associated factors in patients with zero CS and clinical indication for CCTA.

## Methods

### Subjects

This was a cross-sectional, analytical, prospective study carried out from April 2011 to November 2016. Subjects were consecutively and not randomly selected, and subjected to CCTA by referral from their assistant physicians in four diagnostic imaging centers, two public centers: Instituto Dante Pazzanese de Cardiologia de São Paulo-SP e Hospital Universitário do Campus da Saúde Dr. João Cardoso Nascimento da Universidade Federal de Sergipe and two private centers: Hospital Primavera e Clínica de Medicina Nuclear e Diabetes-CLIMEDI.

Data on cardiovascular risk factors were collected from each participant. Chest pain was classified according to the Diamond and Forrester method, and most patients were classified as at intermediate risk for CAD.

Patients with no calcium in the coronary arteries (zero CS) were included. Patients who had undergone percutaneous or surgical myocardial revascularization, patients with history of acute coronary syndrome or cardiomyopathy of ischemic cause, and those who declined to participate were excluded.

The tests performed at private institutions were free of charge for both patients and investigators.

The study was approved by the research ethics committee (CAAE identification number 0289.0.107.000-11).

### CS and CCTA of coronary arteries

CCTA of coronary arteries were performed using a 64-slice (or greater) scanner of the following models and manufacturers: *Aquilion64*™ - Toshiba™ Medical Systems Corporation, Otawara, Japan and Discovery STE VCT - General Electric Company, Connecticut, USA.

Non-contrast computed tomography for CS analysis was carried out using a longitudinal scan coverage from the level of the tracheal bifurcation to the superior border of cardiac silhouette, including the whole diaphragm for evaluation of the whole cardiac area. For CS examination, a field of view (FOV) of 200 mm was used, slice thickness 2.5-3 mm and interval 1.25-1.5 mm, 2 x 32 x 0,6 mm collimaton, rotation time 350 msec, tube current up to 600 mAs.

The study was conducted in two phases: in the first phase, CS was determined by the Agatston score;^6^ calcification was defined as the presence of a lesion with an area greater than 1 mm^2^, and peak intensity equal to or greater than 130 Hounsfield Units (HU), which was automatically identified and marked with color by the software. The presence of coronary plaques and extension of stenosis was evaluated in patients with zero CS.

In the second phase, CCTA was performed using the CS parameters for FOV construction, voltage 120 kv, and 400 miliamperes. Up to 1.5mL/kg iopamidol was administered intravenously to patients still positioned on the table. Iopamidol is a nonionic, iodinated contrast, administered at concentrations of 350-370 mg/mL and rate of 4.5-5.5 mL/s (Ultravist® 370, Bayer HealthCare and Pharmaceuticals, Berlin, Germany; HenetiX® 350, Guerbet, Paris, France).

An oral betablocker was administered within 24 hours before the test, or intravenously on the day of the test in patients with sinus rhythm and heart rate (HR) > 70 bpm. The system uses HR values monitored during the exam to establish the parameters for imaging acquisition, such as the helical pitch (relationship between table distance traveled in one 360º X-ray tube rotation, slice thickness and the number of detector rows), speed of gantry rotation, and exposure time, to achieve the best possible temporal resolution.

Images were sent to the workstation for analysis of coronary arteries by three experienced observers. The presence of atherosclerotic plaque was examined in vessels with a luminal diameter larger than 2 mm, divided into 15 segments.^7^ Extension of stenosis was estimated by calculating the area of the narrowest part of the lumen in relation to the area of the lumen immediately distal to the same segment. Plaques detected by the CCTA were classified into nonobstructive and obstructive lesions, with a reduction ≥ 50% of the lumen in the latter.

### Data analysis

Quantitative variables were described as mean and standard deviation. Kolmogorov-Smirnov test was used to test normality of the sample. The Student’s t-test was used for independent groups, according to data normality. Absolute and relative frequencies were used for categorical variables. For between-group comparisons of these variables, the chi-square test of the Fisher’s exact test was used as appropriate.

Differences were considered statistically significant when probabilities were lower than 5% (p ≤ 0.05) and power of 0.80.

For analysis of independent predictors for the presence of plaque, a manual backwards selection (Backward:Wald method) for logistic regression was used. A p ≤ 0.25 was considered for an initial selection and the variable was maintained in the model when p < 0.05. The outcome variable presence of plaque was adjusted for age, sex, smoking, diabetes mellitus, systemic arterial hypertension, dyslipidemia, family history, obesity and alcohol consumption.

Statistical analyses of results were performed using the SPSS software for Windows version 20.0 (IBM^®^ Corporation, Somers, USA).

## Results

### Clinical characteristics of the sample

In the study period, 1,639 patients were subjected to CCTA at the four participating centers; 619 of them had zero CS. However, 252 were excluded due to lack of clinical data or refusal to participate in the study. Patients were referred to CCTA for atypical chest pain (40.4%), typical chest pain (24.9%), risk factors for CAD, family history of early CAD (51.4%) and positive or inconclusive tests for ischemia (44.4%).

Of 367 patients, 211 (57.7%) patients were hypertensive, 180 (49.3%) dyslipidemic and 55 (15.0%) diabetic. Mean age was 53.7 (±10.5) years and 63.5% were women. Clinical data of patients with zero CS according to the presence or absence of atherosclerotic plaque at CCTA are described in Table 1.

**Table 1:** Clinical characteristics of patients with zero calcium score in diagnostic imaging centers in Sao Paulo and Aracaju, Brazil, from 2001 to 2016

Variable	n^†^	%
Mean age (years) *	367	53.7 ± 10.5
Female sex	233/367	63.5
Systemic arterial hypertension	211/367	57.5
Dyslipidemia	180/367	49.3
Diabetes mellitus	55/367	15.0
Body mass index (kg/m^2^)	316	27.3 ± 4.4
Obesity	77/316	24.4
Family history of CAD	187/364	51.4
Alcohol consumption	135/367	36.8
Smoking	51/366	13.9
Atypical chest pain ^†^	138/342	40.4
Typical chest pain ^†^	85/341	24.9

CAD: coronary artery disease;

(*) Values in mean ± standard deviation; other values expressed as simple frequency (%);

(†) "n" different from total population due to missing data in the records

**Figure 1:**
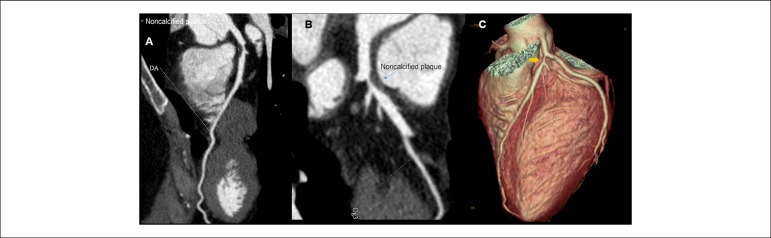
Noncalcified plaque with zero calcium score. Thirty-eight-year old woman; A and B) multiplanar reconstructions showing considerable lumen reduction in anterior descending artery (DA); C) Tridimensional reconstruction showing impairment in DA (yellow arrow).

Frequency of atherosclerotic plaque in coronary arteries was 9.3% (34/367); 95%CI 6.3 - 12.3. In this group, mean age was 52 ± 10 years and 18 (52.9%) were women (Table 2). A detailed analysis revealed the presence of obstructive lesions (larger than 50% of vessel lumen) in 47% (16/34) of cases, distributed as follows: a) in one segment - 12 patients; b) in two segments - 3 patients; and c) in more than two segments - 1 patient (Figure 1). In the subgroup of patients with nonobstructive lesions (18/34), 15 and 3 patients, respectively, had one and three coronary segments affected (Table 3).

**Table 2:** Distribution of clinical characteristics of patients with zero calcium score with and without atherosclerotic plaque in four diagnostic imaging centers in Sao Paulo and Aracaju, Brazil, from 2001 to 2016

Variable	n^†^	With plaque n = 34	Without plaque n = 333	p
Age* (years)	367	52 ± 10.7	53.9 ± 10.5	0.31
Weight (Kg)	367	71.6 ± 12.9	73.7 ± 15.2	0.42
Body mass index (Kg/m^2^)	316	25.9 ± 3.3	27.5 ± 4.4	0.046
Female	233/367	18 (52.9)	215 (64.6)	0.180
Smoking	51/366	8 (24.2)	43 (12.9)	0.073
Non-obese	55/316	29 (90.6)	210 (73.9)	0.037
Diabetes mellitus	55/367	6 (17.6)	49 (14.7)	0.648
Dyslipidemia	180/365	16 (47.1)	164 (49.5)	0.782
Systemic arterial hypertension	211/367	20 (58.8)	191 (57.4)	0.712
Alcohol consumption	135/367	19 (55.9)	116 (34.8)	0.015
Family history	187/364	18 (52.9)	169 (51.2)	0.848

(*) Values as mean ± standard deviation; other values expressed as simple frequency (%); p-value obtained by the chi-square test for associations;

(†) "n" different from total population due to missing data in the records.

**Table 3:** Distribution of atherosclerotic lesions at coronary computed tomography angiography in patients with zero calcium score

Variable	One vessel affected	Two vessels affected	Two or more vessels affected	Total n = 34
Obstructive lesion > 50%	12 (75%)	3 (18.7%)	1 (6.3%)	16 (47.0%)
Nonobstructive lesion	15 (83.3%)	3 (16.6%)	0	18 (53%)

The most affected artery was the anterior descending, 16 (35.56%) in its proximal segment, 10 (22.22%) in the middle segment, and 2 (4.44%) in the distal one.

**Figure 2:**
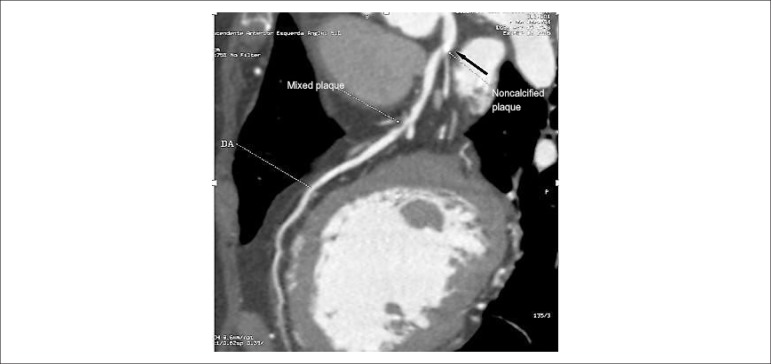
Presence of calcification in zero calcium score. Female patient, 67 years old; Black arrow - Partially calcified plate in anterior descending ostium (AD), not detected by calcium score, followed by noncalcified plaques in proximal and middle thirds (white arrows)

It is worth mentioning that analysis of atheroma in the CCTA with contrast phase revealed that 3/34 (8.8%) patients had plaques with some degree of calcification that were not detected by the CS (Figure 2).

### Clinical features of patients with zero CS, classified by the presence or absence of atherosclerotic plaques in coronary arteries

In patients with coronary artery plaque, most patients were obese (90.6% *vs*. 73.9%; BMI: 25.9 ± 3.3 k/m^2^*vs*. 27.5 ± 4.4 k/m^2^; p = 0.046) and alcohol drinkers (55.9% *vs*. 34.8%) (Table 2). The other variables were not different between the groups.

Non-adjusted odds ratio of the factors associated with the presence of atherosclerotic plaque in patients with zero CS were 2.3 (95%CI = 1.1 - 4.8; p = 0.018) for alcohol consumption and 3.4 (95%CI = 1.0 - 11.5; p = 0.049) for absence of obesity (Table 4). Finally, analysis of contingency table for adjusted odds ratio confirmed higher OR values for alcohol drinkers (OR = 3.4; 95%CI = 1.1 - 5.19; p = 0.018) and non-obese patients (OR = 3.4; 95%CI = 1.0 - 11.7; p = 0.047) (Table 5).

**Table 4:** Factors associated with the presence of plaque^†^ (diagnostic imaging centers in Sao Paulo and Aracaju, Brazil, from 2001 to 2016)

Variable	Non-adjusted odds ratio	95%CI	p
Age	0.976	0.940 - 1.01	0.216
**Sex**			
Male	1.62	0.796- 3.29	0.183
**Smoking**			
Yes	2.15	0.919 - 5.09	0.079
**Obesity**			
No*	3.40	1.01 - 11.51	0.049
**Diabetes mellitus**			
Yes	1.24	0.489 - 3.15	0.649
**Dyslipidemia**			
Yes	1.10	0.545 - 2.24	0.782
**Systemic arterial hypertension**			
Yes	1.06	0.519 - 2.17	0.869
**Alcohol consumption**			
Yes	2.37	1.16 - 4.83	0.018
**Family history**			
Yes	1.07	0.528 -2.17	0.366

Outcome variable: presence of plaque; other variables described in the table are associated factors;

(*) presence of obesity was used as reference for the variable obesity, CI: confidence interval;

(†) adjusted for age, sex, smoking, diabetes mellitus, systemic arterial hypertension, dyslipidemia, family history, obesity and alcohol consumption.

**Table 5:** Factors associated with the presence of plaque^†^ after model adjustment in diagnostic imaging centers in Sao Paulo and Aracaju, Brazil, from 2001 to 2016

Variable	Adjusted odds ratio	95%CI	p
Alcohol consumption	3.46	1.16 - 5.19	0.018
Non-obese*	3.45	1.01 - 11.7	0.047

Outcome variable: presence of plaque; other variables described in the table are associated factors;

(*) presence of obesity was used as reference for the variable non-obese, CI: confidence interval

(†) adjusted for age, sex, smoking, diabetes mellitus, systemic arterial hypertension, dyslipidemia, family history, obesity and alcohol consumption.

## Discussion

The main finding of the present study was the considerable presence (9.3%) of obstructive (≥ 50%) coronary atherosclerotic plaques in patients with zero CS.

Clinical features found to be associated with the presence of plaques were alcohol consumption and absence of obesity, in contrast to other risk factors usually associated with CAD, such as: diabetes mellitus, systemic arterial hypertension and dyslipidemia.^8^

Data on the literature have shown variable prevalence of atherosclerotic plaque in individuals with zero CS. In a study conducted in Isfahan (Iran), 385 patients with zero CS were studied, and 16 of them (4.1%) had atherosclerotic plaque at CCTA.^5^ In another study involving symptomatic and asymptomatic patients showed that only symptomatic subjects with zero CS had atherosclerotic plaque (8.4%).^9^ According to the CONFIRM study, in patients with zero CS, 13% had nonobstructive atherosclerotic lesions, and 3.5% had obstructive lesion greater than 50%.^4^ A multicentric cohort study in which Brazil participates (a CORE64 sub-study) confirmed that a zero CS does not exclude the need for revascularization. With a sample of 291 patients (72 with zero CS), 19% had stenosis ≥50%, and 13% of them required revascularization.^10^

Also, studies involving patients with chest pain in the emergency department have shown frequencies of atherosclerotic plaques with zero CE of up to 39%,^11^^-^^13^ although this is a different population from those attending outpatient services. It is of note, however, that our sample population was composed of patients referred to CCTA from their assistant physicians. As reported in international studies, we also found that the presence of atherosclerotic plaque cannot be ruled out in patients with zero CS.

In our study, only the variables alcohol consumption and absence of obesity were associated with higher risk of atherosclerotic plaque, in contrast to classical risk factors for CAD (diabetes mellitus, systemic arterial hypertension and dyslipidemia). Interestingly, higher BMI was associated with absence of atherosclerotic lesion. Previous studies have suggested obesity as a protective factor for CAD, the so-called obesity paradox.^14^ Nevertheless, such paradox is not concerned to abdominal obesity, which has been associated with CAD and considered more pathological than subcutaneous fat accumulation.^14^^-^^16^ In our study, we did not measure abdominal circumference, which may have influenced the consistency of results. Besides, obese patients included in many studies that indicated obesity as a protective factor were younger, which may be a source of bias.^17^

Alcohol consumption has also yielded diverging results. While some studies have indicated alcohol consumption as a risk factor for CAD, others have pointed out its beneficial effects, such as studies performed with wine and its component resveratrol.^18^^-^^20^ Resveratrol is known for its antioxidant and anti-inflammatory effects, in addition to promote the synthesis of HDL in the liver and inhibit LDL production, thereby preventing LDL oxidation and reducing the risk of cardiovascular diseases.^21^ In this regard, further studies that specify the type of beverage consumed and not only whether the subjects consumed or not alcohol are needed.

### Limitations

Some inherent limitations deserve to be mentioned - first, as previously described, patients were referred to CCTA with CS from their assistant physicians, and the possibility of a selection bias cannot be excluded; second, coronary risk stratification of patients was not performed before their inclusion and data on risk factors were obtained by questionnaires; third, sample was collected in four different centers and, although the tests were performed following similar protocols, some characteristics are particular of each service which may have cause a bias in the analysis; fourth, since we studied patients with clinical indication for CCTA, our sample differed from asymptomatic patients without positive ischemic test, who would be referred to CS alone, and in whom coronary calcification would predict cardiovascular events.

## Conclusions

The frequency of atherosclerotic plaque in patients with zero CS was relatively high, indicating that in patients with clinical indication for CCTA, the absence of coronary calcification does not exclude atherosclerotic plaque or obstructive lesion, especially in obese and alcohol drinkers.
